# Supplementary material for: Divergent venom effectors correlate with ecological niche in neuropteran predators
Source: Commun Biol. 2024 Aug 13;7:981. doi: 10.1038/s42003-024-06666-9 (PMC11319779; doi:10.1038/s42003-024-06666-9)
Supplement: Supplementary file 2 — Supplementary Information [file 42003_2024_6666_MOESM2_ESM.pdf]

Supplemental Information for:

Divergent venom effectors correlate with ecological niche in neuropteran predators

Maike Laura Fischer, Henrike Schmidtberg, Olivia Tidswell, Benjamin Weiss, Ludwig Dersch, Tim Lüddecke, Natalie Wielsch, Martin Kaltenpoth, Andreas Vilcinskas, Heiko Vogel

Supplementary figures:

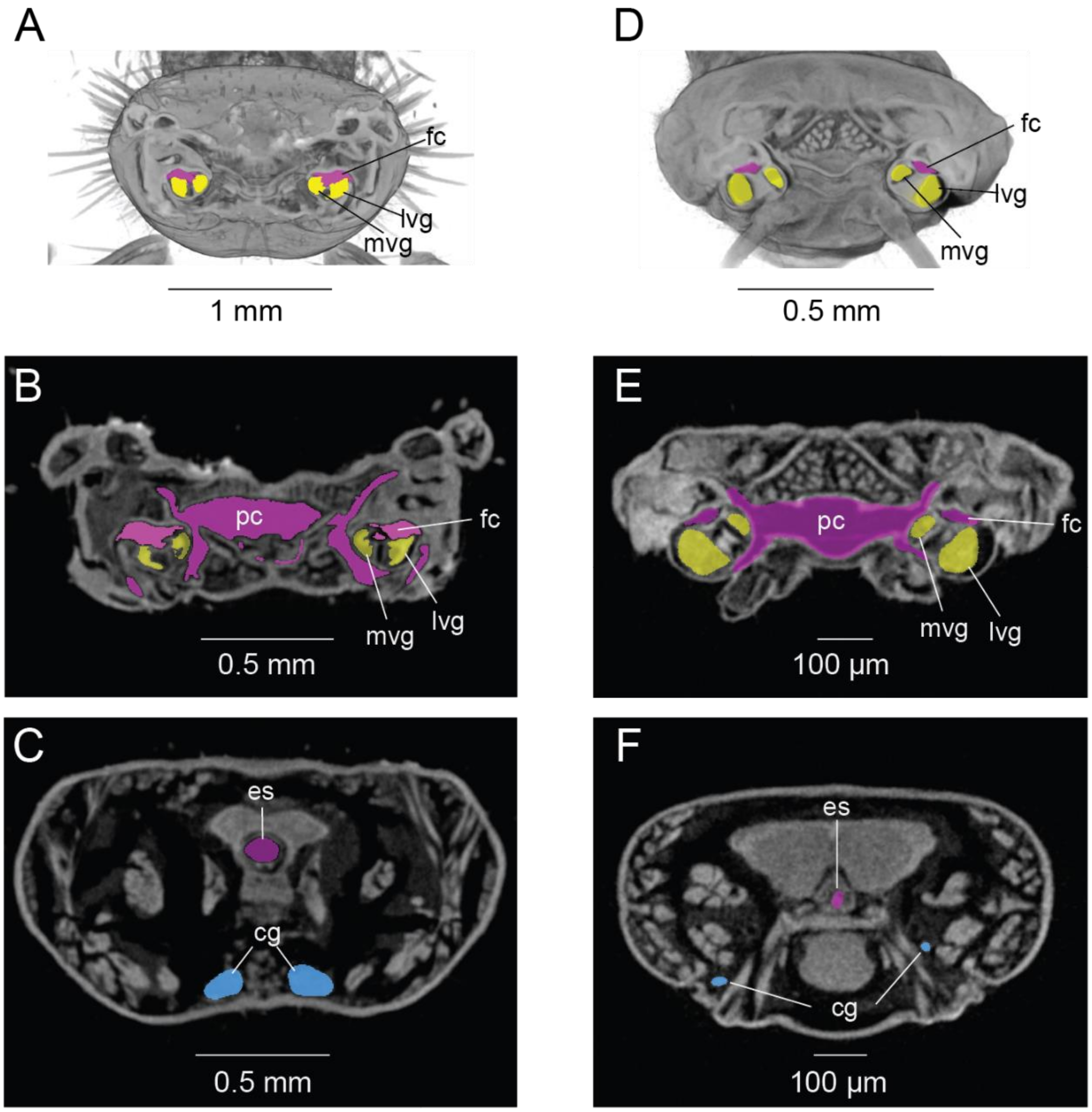

Figure S1: Reconstructions of the venom system and digestive tract of *Euroleon nostras* (**A**, **B**, **C**) and *Chrysoperla carnea* (**D**, **E**, **F**) using micro-computed tomography. **A**, **B**, **D**, **E**: Transversal view on the medial venom gland (mvg) and lateral venom gland (lvg) and the digestive tract including

the food canal (fc) and the preoral chamber (pc). The cephalic glands (cg) extend posteriorly into the head to where the esophagus (es) is located (**C**, **F**).

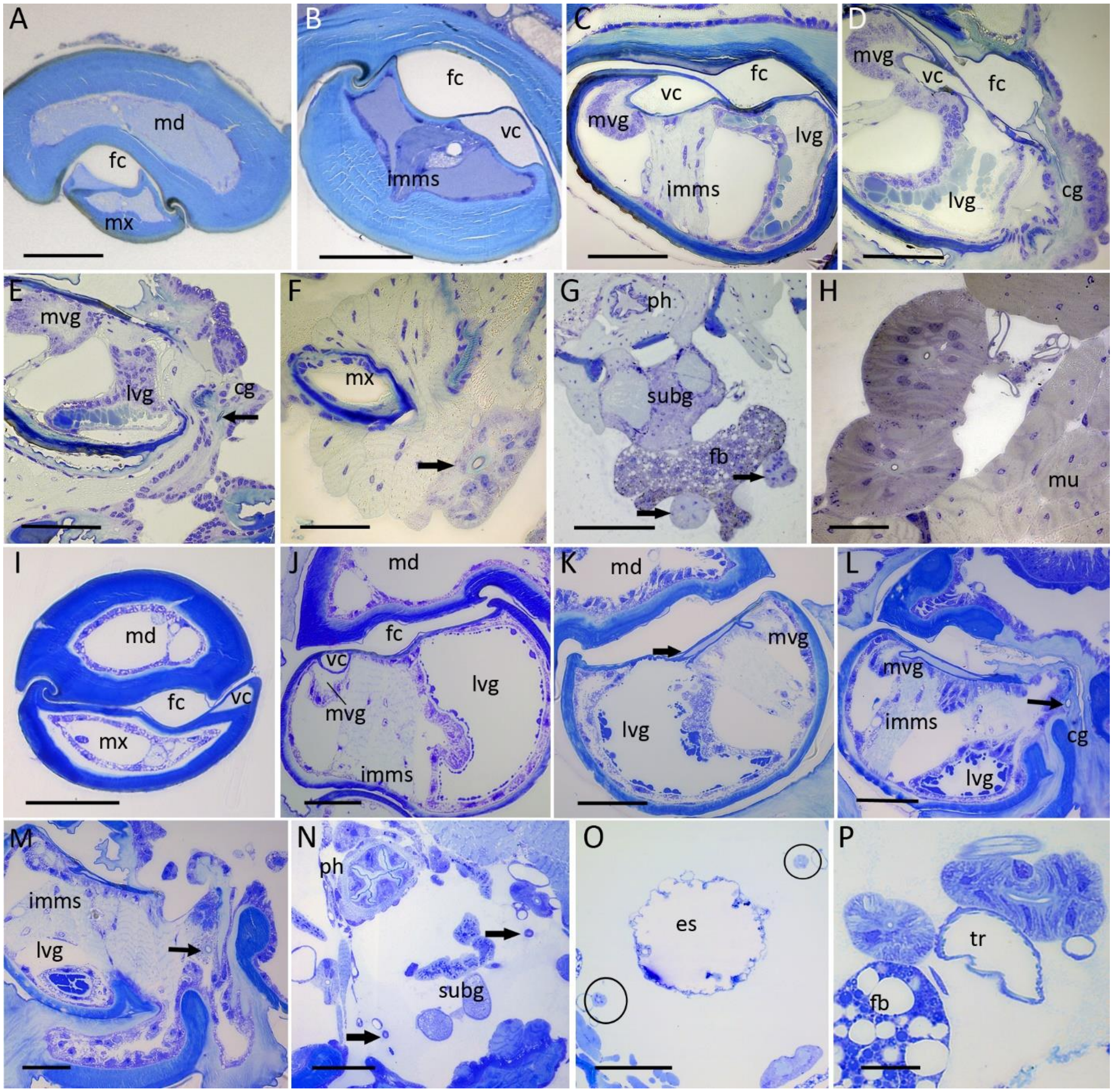

Figure S2: Putative venom system of *Euroleon nostras* (**A** - **H**) and *Chrysoperla carnea* (**I** - **P**). **A**: Transverse section of interlocking mandible (md) and maxilla (mx) at distal region of the stylets of *E. nostras* forming the food canal (fc), scale bar 50 µm. **B**: In the distal region of the maxilla, the venom canal (vc) clearly protrudes into the food canal. Intrinsic muscles of the maxillary stylet (imms) are directly connected to the venom canal, scale bar 50 µm. **C**: At the base of each maxilla, separated by the intrinsic muscle of the maxillary stylet and connected to the venom canal, are two distinct glandular structures. These glands are located within the maxillae forming the medial

venom gland (mvg) and the lateral venom gland (lvg), scale bar 100 µm. **D**: Large glandular cells of the cephalic gland (cg, arrow) are located at the base of the food canal, scale bar 100 µm. **E**: The cells of both cephalic glands form tubular extensions with a cuticula-lined ductus (arrow) which protrude along the alimentary system posterior into the head, scale bar 100 µm. **F**: Magnification of the cephalic gland (arrow) with its ductus at the most basic part of the maxilla, scale bar 100 µm. **G**: Transverse section of the pharynx (ph) and the sub-esophageal ganglion (subg) with adjacent fat body tissue (fb) and the two tubular cephalic glands (arrows), scale bar 200 µm. **H**: Magnification of the transverse section through the cephalic glands with their cuticular ductus close to muscular tissue (mu), scale bar 50 µm. **I**: Transverse section of interlocking mandible and maxilla at distal region of the stylets of *C. carnea*. In contrast to the stylets in *E. nostras*, the mandible and maxilla of *C. carnea* have almost the same size and the venom canal is proportionally smaller, scale bar 50 µm. **J**: At the base of each maxilla the intrinsic muscle of the maxilla separates the medial venom gland and the lateral venom gland, scale bar 50 µm. **K**: Both, the medial venom gland and the lateral venom gland are in contact with the venom canal (arrow), scale bar 50 µm. **L**: At the base of the food canal glandular cells of the cephalic glands are visible and form a ductus (arrow), scale bar 50 µm. **M**: The cephalic gland (arrow) proceeds laterally and subsequently ventrally in the head along the alimentary system, scale bar 50 µm. **N**: Transverse section of the pharynx, margin of sub-esophageal ganglion, and the two tubular cephalic glands (arrows), scale bar 100 µm. **O**: Cephalic glands (encircled) at the level of the esophagus (es), where they disappear after a few further sections, scale bar 200 µm. **P**: Magnification of the transverse section through the cephalic glands with their cuticular ductus close to tracheoles (tr), scale bar 25 µm.

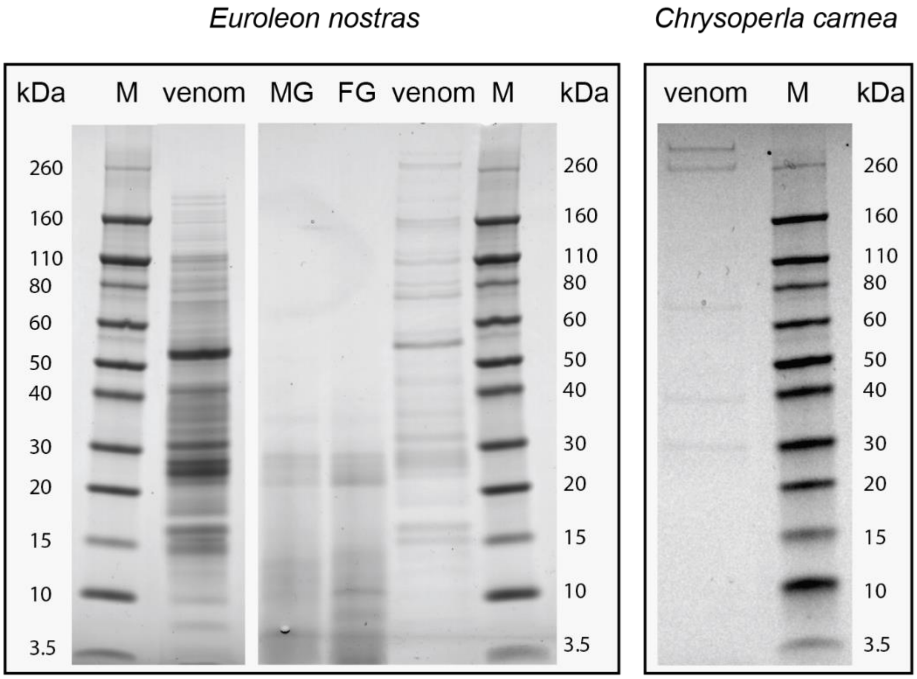

Figure S3: SDS-PAGE analysis of venom collected from *Euroleon nostras* and *Chrysoperla carnea* using a prey dummy and *E. nostras* gut extracts. FG = foregut extract; MG = midgut extract; M = protein marker; venom = prey dummy venom (*E. nostras*: two pooled samples from different collections).

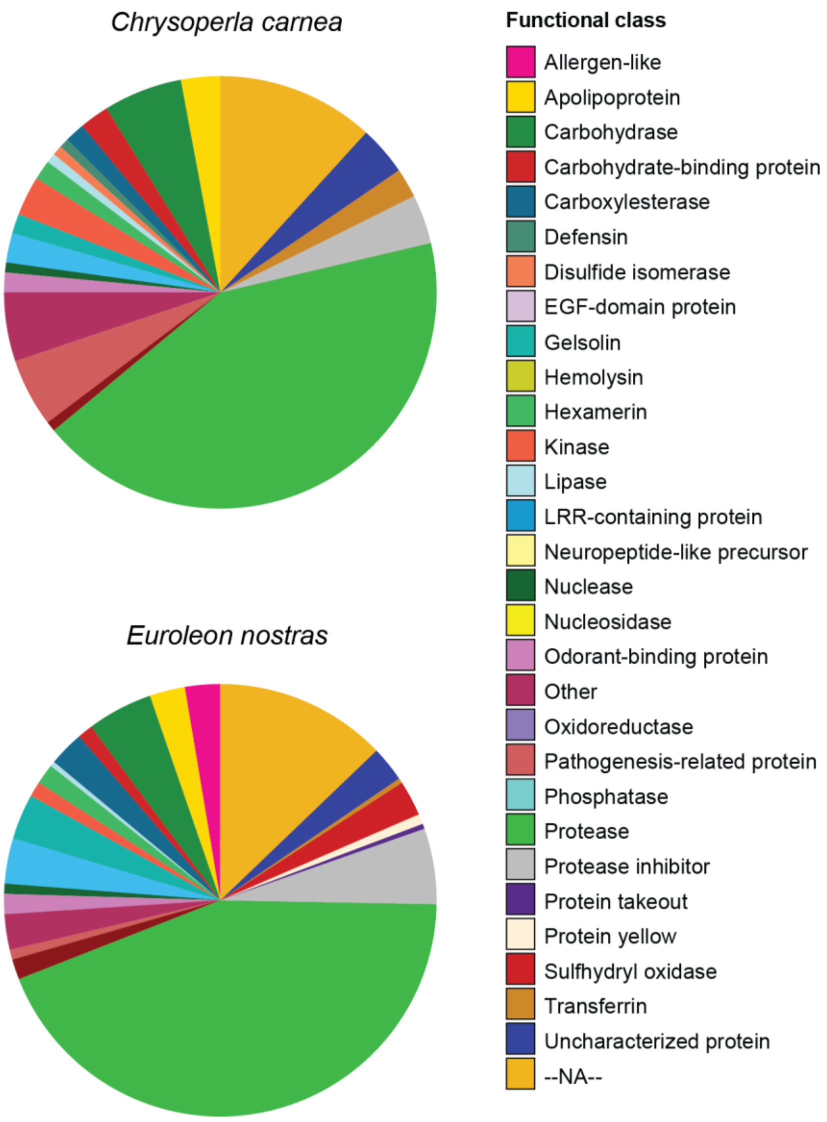

Figure S4: Venom proteins identified in the proteome of *Euroleon nostras* and *Chrysoperla carnea*. The identified proteins were grouped according to their protein family membership associations represented by color-coded blocks.

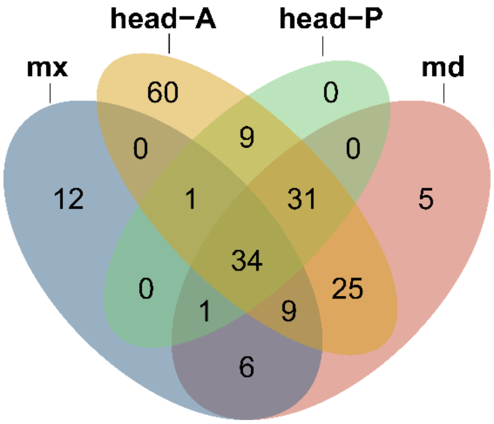

Figure S5: Tissue-specific expression of strongly expressed proteins (log_2(TPM+1) ≥ 5) that were detected in the venom proteome of *Euroleon nostras*.

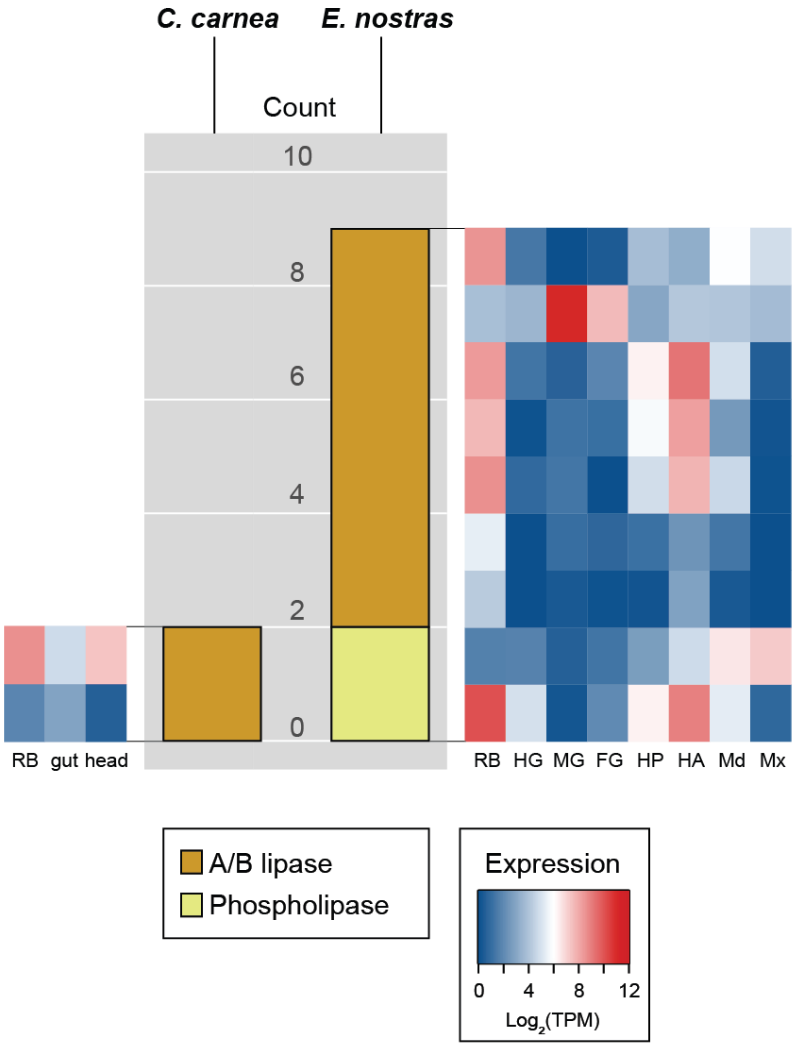

Figure S6: Expression of lipases identified in the proteome of *Euroleon nostras* and *Chrysoperla carnea*. The heat maps represent the relative expression levels ($\log_2$(TPM)) in the maxillae (Mx), mandibles (Md), anterior head (HA), posterior head (HP), foregut (FG), midgut (MG), hindgut (HG), and remaining body tissue (RB) of *E. nostras*, and in the head, gut and RB of *C. carnea*. Color-coded blocks represent the classification into alpha-beta fold lipases (A/B lipase) or phospholipases.

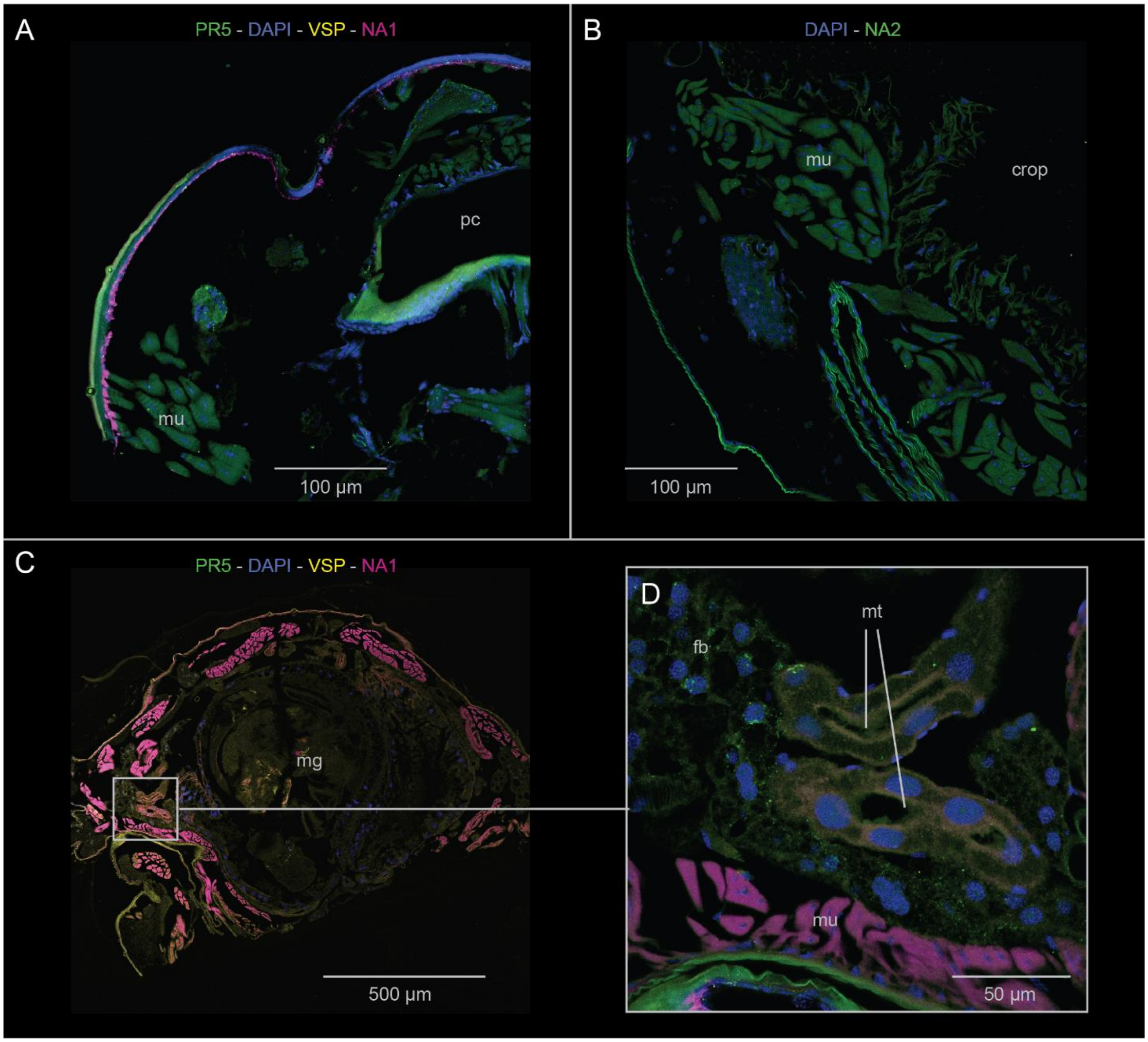

Figure S7: Expression of selected putative venom protein encoding genes in *Euroleon nostras*. Cross sections were hybridized with gene-specific hybridization chain reaction RNA fluorescence *in situ* hybridization (HCR-RNA-FISH) probes for (**A**) *pr-5*, *vsp*, *na1* and (**B**) *na2*. Host nuclei are shown in blue. *Pr-5* is weakly expressed throughout tissues of the head (**A**) and abdomen (**C**), including the fat body (fb) and malpighian tubules (mt) (**D**). *Na1* is strongly expressed in epithelial cells (**A**). *Na2* (**B**) and vsp (**A**, **C**, **D**) are not expressed in non-venom related tissues. pc = pre-oral chamber; mu = muscle.

-21 -1 1
MSNFFKLLFTVLCVVVVYTNA|IVNDLKNSSSLIPEHKEDKKDQQETPIVSRGSFQDDY

60
YQHRQIQHKTYKIYRCTYPVRR**SQQKQYPHDHGQHPRDQEQYPYDKRQYPHDQEQ**

HPRDKGQYPRDQEQYPYDKGQYPHDQEQYPHDKGQHPRDQEQYPHDKGQYPQD

158
KRQYPHIQGKYPYSAYIGDEKTPNELPKESMDFVNSPIIDYRFCKFIGAVTDSDDMPPS

258
PIPSPDPLPRQGHDYQRMDRGRVPSVDHSRYPISGHRPRIPTSYIPSFGADFFV

Figure S8: Full amino acid sequence of the uncharacterized venom protein NA2 from *Euroleon nostras*. The signal peptide is highlighted in italics and the vertical line indicates the cleavage site. Tandem repeats with conserved proline residues are highlighted in bold and yellow, respectively.

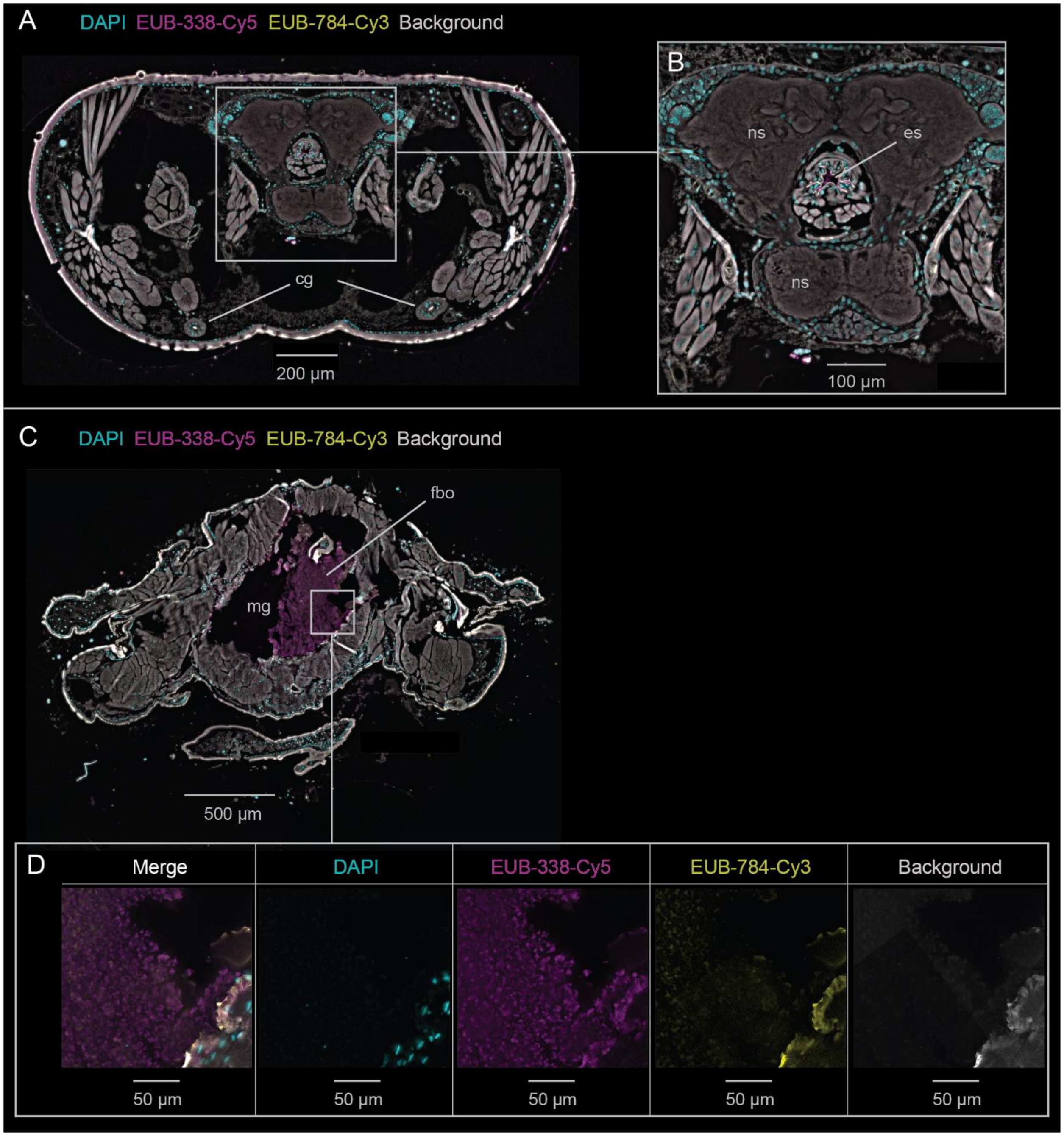

Figure S9: Detection of bacteria in semithin transverse histological sections of *Euroleon nostras* using fluorescence *in situ* hybridization. The fluorescently labelled eubacterial probes EUB-338-Cy5 (magenta) and EUB-784-Cy3 (yellow) were used alongside a DAPI stain (cyan). *E. nostras* harbors no bacteria in any tissue of the head (**A**) and thorax (**C**), including the cephalic gland (cg), esophagus (es) (**B**) and midgut (mg) (**C**). The food bolus (fbo) in the mg contains autofluorescent substances but no bacterial cells (**D**). ns = nervous system.

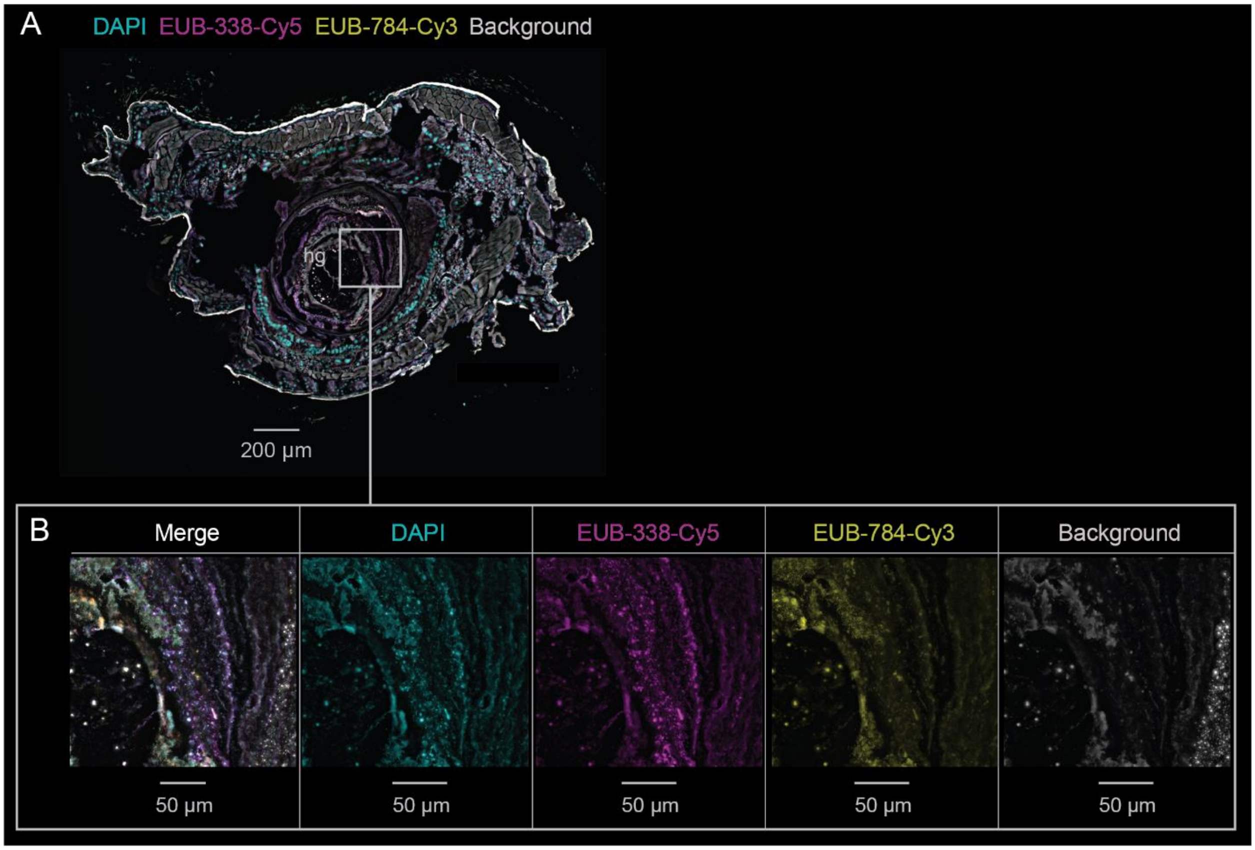

Figure S10: Detection of bacteria in semithin transverse histological sections of *Euroleon nostras* using fluorescence *in situ* hybridization. The fluorescently labelled eubacterial probes EUB-338-Cy5 (magenta) and EUB-784-Cy3 (yellow) were used alongside a DAPI stain (cyan). *E. nostras* harbors no bacteria in the hindgut (hg) (**A**). The food bolus (fbo) in the hg contains autofluorescent substances but no bacterial cells (**B**).

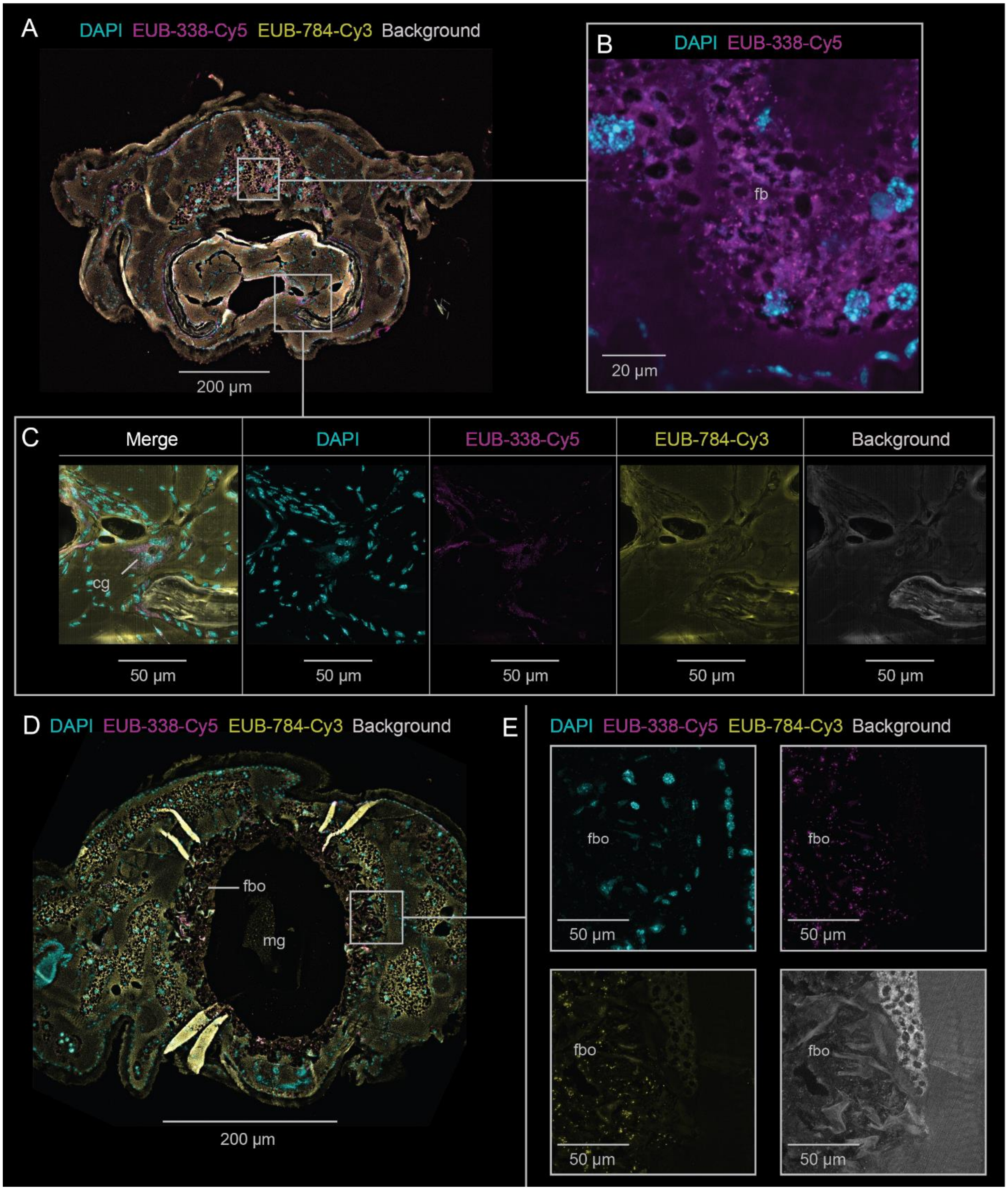

Figure S11: Detection of bacteria in semithin transverse histological sections of *Chrysoperla carnea* using fluorescence *in situ* hybridization. The fluorescently labelled eubacterial probes EUB-338-Cy5 (magenta) and EUB-784-Cy3 (yellow) were used alongside a DAPI stain (cyan). *C. carnea* harbors bacteria in tissues of the head (**A**) including the fat body (fb) (**B**) and cephalic gland (cg) (**C**)**.** The food bolus (fbo) in the midgut (mg) contains bacterial cells (**D**, **E**).

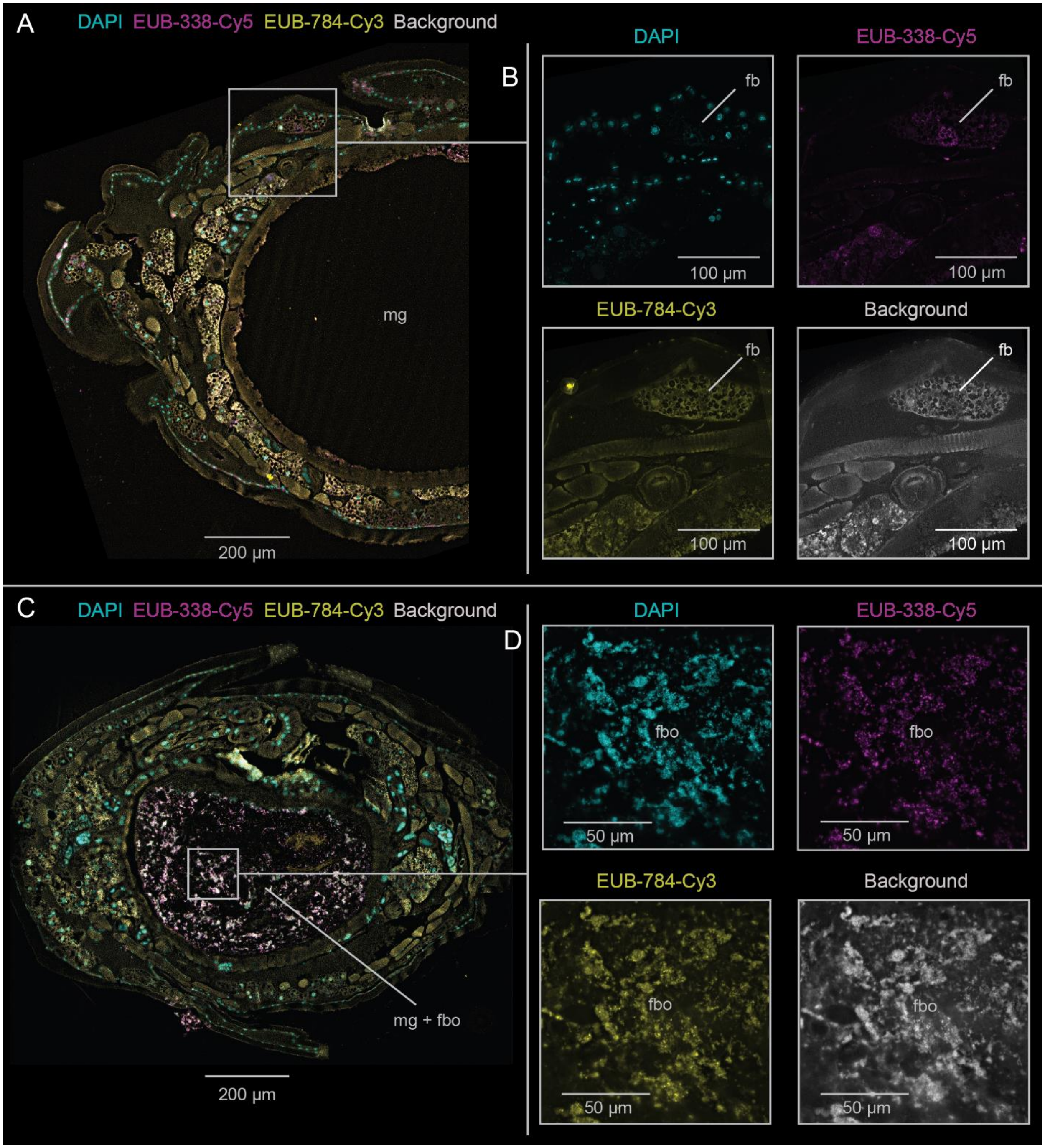
A
DAPI EUB-338-Cy5 EUB-784-Cy3 Background
mg
200 µm
B
DAPI
EUB-338-Cy5
EUB-784-Cy3
Background
fb
100 µm
C
DAPI EUB-338-Cy5 EUB-784-Cy3 Background
mg + fbo
200 µm
D
DAPI
EUB-338-Cy5
EUB-784-Cy3
Background
fbo
50 µm

Figure S12: Detection of bacteria in semithin transverse histological sections of *Chrysoperla carnea* using fluorescence *in situ* hybridization. The fluorescently labelled eubacterial probes EUB-338-Cy5 (magenta) and EUB-784-Cy3 (yellow) were used alongside a DAPI stain (cyan). *C. carnea* harbors bacteria in the fat body (fb) (**A**, **B**). The food bolus (fbo) in the midgut (mg) contains a high denisity of bacterial cells (**C, D**).

Supplementary Tables:

Table S1: Summary of *de novo* reference transcriptome assemblies from *Euroleon nostras* and *Chrysoperla carnea*.

Species	Tissue	Number of reads (Mio.)	Number of contigs	N50 contig size	BUSCO coverage (Insecta_odb9 database)
E. nostras	RB	38.3	58.825	1.453	C:86.0%[S:83.6%,D:2.4%],F:7.0%,M:7.0%
	HG	66.5			
	MG	62.8			
	FG	71.9			
	Head-P	75.3			
	Head-A	72.7			
	Md	73.5			
	Mx	71.8			
C. carnea	RB	41.9	65.221	2.070	C:89.3%[S:88.2%,D:1.1%],F:4.8%,M:5.9%
	Gut	38.9			
	Head	45.1			

Table S2: Putative bacterial proteins detected by LC-MS/MS in the venom proteome of *Euroleon nostras*.

Protein	Organism	Protein Description	Total Peptides	Unique Peptides	Total Intensity
sp|Q24800|SEVE_ECHGR	*Echinococcus granulosus*	Severin	1	1	1.73E+08
sp|B9JQW4|G6PI_AGRVS	*Agrobacterium vitis*	Glucose-6-phosphate isomerase	1	1	1.58E+08
sp|A5CU93|KAD_CLAM3	*Clavibacter michiganensis* subsp. *michiganensis*	Adenylate kinase	1	1	9.65E+07

sp|C0ZVT7|EFTU_RHOE4	*Rhodococcus erythropolis*	Elongation factor Tu	1	1	0
sp|A6L8N5|ATPA_PARD8	*Parabacteroides distasonis*	ATP synthase subunit alpha	1	1	0

Table S3: Putative bacterial proteins detected by LC-MS/MS in the venom proteome of *Chrysoperla carnea*.

Protein	Organism	Protein Description	Total Peptides	Unique Peptides	Total Intensity
sp|Q983S4|CH604_RHILO	*Mesorhizobium japonicum*	Chaperonin GroEL 4	9	1	7.73E+07
sp|B9K1Y8|CH60_AGRVS	*Agrobacterium vitis*	Chaperonin GroEL	4	1	7.73E+07
sp|P24753|G3P_SEROD	*Serratia odorifera*	Glyceraldehyde-3-phosphate dehydrogenase (Fragment)	3	1	6.71E+07
sp|Q2NUI3|DPS_SODGM	*Sodalis glossinidius*	DNA protection during starvation protein	3	3	6.02E+07
sp|Q98F85|PAL_RHILO	*Mesorhizobium japonicum*	Peptidoglycan-associated lipoprotein	4	3	5.22E+07
sp|Q2NRD5|PGK_SODGM	*Sodalis glossinidius*	Phosphoglycerate kinase	3	1	2.55E+07
sp|B1MGH7|EFTU_MYCA9	*Mycobacteroides abscessus*	Elongation factor Tu	2	1	1.02E+07
sp|Q11HA6|EFTU_CHESB	*Chelativorans* sp.	Elongation factor Tu	2	1	7.00E+06
sp|Q98GS0|IHFB_RHILO	*Mesorhizobium japonicum*	Integration host factor subunit beta	1	1	6.46E+06
sp|Q8UGF0|RL9_AGRFC	*Agrobacterium fabrum*	50S ribosomal protein L9	2	1	6.07E+06
sp|P23847|DPPA_ECOLI	*Escherichia coli*	Dipeptide-binding protein	1	1	3.29E+06

sp|Q8ZB98|IPYR_YERPE	*Yersinia pestis*	Inorganic pyrophosphatase	1	1	3.15E+06
sp|Q89AJ7|ODO1_BUCBP	*Buchnera aphidicola* subsp. *Baizongia pistaciae*	Oxoglutarate dehydrogenase	1	1	2.56E+06
sp|Q0W1V5|RS4_METAR	*Methanocella arvoryzae*	30S ribosomal protein S4	1	1	2.32E+06
sp|Q2NWS0|RL11_SODGM	*Sodalis glossinidius*	50S ribosomal protein L11	1	1	1.96E+06
sp|P50204|PHAB_PARDE	*Paracoccus denitrificans*	Acetoacetyl-CoA reductase	1	1	1.72E+06
sp|P29272|G3P_CERSP	*Cereibacter sphaeroides*	Glyceraldehyde-3-phosphate dehydrogenase	1	1	1.66E+06
sp|P53573|ETFA_BRADU	*Bradyrhizobium diazoefficiens*	Electron transfer flavoprotein subunit alpha	1	1	1.62E+06
sp|Q2NW23|IF2_SODGM	*Sodalis glossinidius*	Translation initiation factor IF-2	1	1	7.45E+05
sp|Q1GVQ9|RS15_SPHAL	*Sphingopyxis alaskensis*	30S ribosomal protein S15	1	1	6.15E+05
sp|Q49842|MIAB_MYCLE	*Mycobacterium leprae*	tRNA-2-methylthio-N(6)-dimethylallyladenosine synthase	1	1	0.00E+00
sp|Q2NRG1|SYK_SODGM	*Sodalis glossinidius*	Lysine--tRNA ligase	1	1	0
sp|Q98DU8|SECB_RHILO	*Mesorhizobium japonicum*	Protein-export protein SecB	1	1	0
sp|Q2S6J0|RS2_SALRD	*Salinibacter ruber*	30S ribosomal protein S2	1	1	0

Table S4: BLAST search results of *Euroleon nostras* venom proteins against the *Chrysoperla carnea* genome (GenBank accession GCA_905475395.1). Only venom proteins present in the *E. nostras* proteome and without known homologs were used as queries. The e-value cut-off was set to 0.01.

Contig name	E-value	Homolog in *C. carnea* genome	Homolog in *C. carnea* proteome
Enos_GHB_C1395	-	-	
Enos_GHB_C15574	-	-	
Enos_GHB_C16334	-	-	
Enos_GHB_C16335	-	-	
Enos_GHB_C18080	-	-	
Enos_GHB_C18774	1.75E-11	FR997756.1	Ccarn_LC_C12479
Enos_GHB_C21181	-	-	
Enos_GHB_C21182	-	-	
Enos_GHB_C26697	-	-	
Enos_GHB_C27040	5.31E-16	FR997754.1	
Enos_GHB_C27381	-	-	
Enos_GHB_C27382	-	-	
Enos_GHB_C28807	-	-	
Enos_GHB_C32114	1.12E-09	FR997756.1	Ccarn_LC_C12479
Enos_GHB_C37675	-	-	
Enos_GHB_C38003	-	-	
Enos_GHB_C3910	-	-	
Enos_GHB_C40207	-	-	
Enos_GHB_C40776	-	-	
Enos_GHB_C40777	-	-	
Enos_GHB_C41454	-	-	
Enos_GHB_C42125	-	-	
Enos_GHB_C43272	-	-	
Enos_GHB_C43420	-	-	
Enos_GHB_C45094	0.001	FR997754.1	
Enos_GHB_C46612	-	-	
Enos_GHB_C47276	6.63E-25	FR997754.1	Ccarn_LC_C9682
Enos_GHB_C51622	-	-	
Enos_GHB_C52278	0.002	FR997757.1	
Enos_GHB_C52358	-	-	
Enos_GHB_C7690	-	-	
Enos_GHB_C7755	-	-	
Enos_GHB_C9218	-	-	

Enos_GHB_C9242	-	-	

Table S5: Full coding sequences and corresponding contig names of the genes *pr-5*, *vsp*, *na1* and *na2* from *Euroleon nostras* that were used for hybridization chain reaction RNA fluorescence *in situ* hybridization.

Gene name	Contig name in the transcriptome	Full coding sequence
pr-5	Enos_GHB_C137	ATGACAACAATCAGTATTTTAGTGTCGTTTGTTATTTTAACGGTTTATGTTGTTAACGGTCG<br>AGAATTTCAATTTTTGAATAATTACGGACAACAATTATGGCTCGGAATACAAGGAAATAGT<br>GGAAAAGGGACACCAAATGGCGGTGGATTTGTATTAAATCCTGGACAAAGATCATCCATA<br>CATGTTGCTGACGATTGGGGTGGTCGTTTTTGGGCAAGAACCGGTTGCAATGGCGGTAAT<br>AATCATTGTGAAACAGGCGATTGTGGCAATCGTTTAGAGTGCGGAGGTAATGGTGGTGCA<br>CCTCCCGTCAGTTTAGCTGAAATAACCCTGAAAGGATGGGGAGGCCTTGATTATTATGACC<br>TTTCATTGGTCGATGGTTTCAATATACCAATTGCTATGGAACCATTAGGTGGACAAGGTGA<br>TGGTAGTCAATATAGTTGTAAAAGAGCCACATGCCATGCAAATATAAATGGTGATTGTCCA<br>AATGAATTACGTTTATGGTCGAACGGGAATGTAGTTGGATGTAAATCAGCTTGTTTAGCAT<br>TCAATACGGATCAATATTGTTGCCGTGGTGCACATAATCGTCCAGAAACATGTCGGTCAAG<br>TGATTGGCCGGTTAATTATCCACGATGGTTTAAAGATCGTTGTCCAGATGCATACAGTTAC<br>GCTTACGATGATCATAAGAGTACATTTACATGTCGTGCACCAGCATATTTGGTGACCTTCG<br>GTTAA
vsp	Enos_GHB_C52324	ATGGATCCGCGACTCACTAGTCTATTTGTATTATTAATATTTTGTTGTGGACAAATTACAGC<br>AAATGAAAAATGTACAACACCTGATGCTGATCCCGGCGAATGTGTTGATGTGGACAATTGT<br>CCACAACTGAAATCGGCACTGTATCGTATCTATGGCCAGGATGCCATACAACATATGGAAC<br>GTGATGTGCACGTAAACATGTCTACGTGTGGATGGAATGATGAACGTGATGTGCGGAAGG<br>TGTGTTGTCCCACAGAAACTAGCGCCGATATGGATACCGATGCTATGTCAATGTTTGGCGA<br>ACCGGAAGGAACGTTTGAACTAGCCAATCCGGGAGTATGTGGTGTTACGTCAGCTAATGA<br>TTCATACTCACGTATTGTTGGCGGTTTTACTGTTAAAAAAGGACAATTTCCATGGATTGTTG<br>CGTTGGGATATGACGAAGGGAATATTTACTATCCAGACTGGAAATGTGGAGGTTCCTTAAT<br>TACACCAAATCATGTATTAACAGCTGCTCATTGTGCATTACCAAACTTAATCAAAGTTAAAA<br>TTGGAACCCTGAATCCATGGGCACCTGGATATTTAGTCGGCGATGTTATCGAAATCCACAA<br>ACACGAAAAATATGATAACGTGACGGATGTTTTCGATATTGCAGTTTTGAAAATTTCATGG<br>AGAGATTATTATGGCAAACCAACAGAATTTAAACCATCAGATGAAGCCCGACTAATCTGTT<br>TGCCGAAAGAAATGGATATGCGCAAAAAATCGTATGAGGGATACTTTCCATTTGTGGCTG<br>GATGGGGCGTTATGGAATGGAATACAACAGACACTAGCACCGATTTATTAGCTGTACAAG<br>TTAAAATTGTTAAACAGAGTATTTGTCGTAAGAAATATGCAAATTTCAAACGTATTAAGGT<br>GACAAATAATGTAATGTGTGCCGGATATAAAACCGATCGTTTGGATTCGTGTCAAGGTGAT<br>TCTGGTGGTCCATTAATGTTACCACATTTAGGTCCAGACGGTCTGATCTATTATTATCAAAT<br>TGGTATTGTGTCGAAAGGTTATCGATGTGCCACTAAAGGGATGCCAGCCGTTTATACGCGT<br>GTCGGTAACTTTATTGAATGGATTAATGCAAAGATTAATTTATAG
na1	Enos_GHB_C1012	ATGTTCTACAAACTTTTCCTCATCTGTCTTATTGCTTTGATCGCTGTTGCCACAGCTGATCCA<br>CAATTATACTACAGCGGATATTATCCAGGTGCCCGTGCCTATGCTGGTGGCTATTACCCATA<br>TGCCGGTTACCCAGCCGTAGCTTATTATGGCAAGTGA
na2	Enos_GHB_C9242	ATGTCCAATTTTTTTAAATTGTTGTTCACAGTATTATGTGTTGTTGTGGTTTATACGAATGCA<br>ATTGTGAATGACCTTAAAAATTCTTCATCGCTCATACCGGAACATAAGGAAGATAAAAAGG<br>ATCAACAAGAAACTCCTATAGTTTCACGAGGATCATTCCAAGACGATTATTACCAACATCGA<br>CAAATACAACATAAAACATACAAAATTTATCGATGCACGTATCCTGTTCGACGTAGTCAACA<br>GAAACAGTATCCTCACGATCATGGGCAGCACCCTCGCGATCAGGAACAATACCCTTACGAC<br>AAAAGACAGTATCCTCACGATCAAGAGCAGCACCCCCGTGATAAAGGGCAGTATCCTCGG<br>GATCAGGAACAGTACCCCTATGACAAAGGACAGTACCCTCATGATCAAGAACAGTATCCCC<br>ACGATAAAGGGCAGCATCCTCGGGATCAGGAACAATACCCTCACGATAAAGGACAGTACC<br>CACAAGATAAAAGACAATACCCACATATACAAGGAAAATATCCGTATTCAGCATATATAGG<br>TGATGAAAAAACACCAAATGAATTACCAAAAGAATCAATGGACTTCGTTAACTCACCAATA<br>ATTGATTATCGATTTTGCAAATTTATTGGTGCCGTTACGGATTCTGATGATATGCCTCCATCT<br>CCTATCCCTTCACCAGATCCATTACCAAGACAAGGACATGATTATCAACGTATGGATCGTG

		GCCGCGTTCCATCTGTGGACCATTCACGTTATCCAATTAGTGGTCATCGTCCACGAATACCT ACTAGTTATATACCATCATTCGGTGCGGATTTTTTTGTATAG

Table S6: Results of the BUSCO analysis for the transcriptome assemblies of *Euroleon nostras* and *Chrysoperla carnea* and the genome assembly of *E. nostras*. C = complete BUSCOs, S = complete and single-copy BUSCOs, D = complete and duplicated BUSCOs, F = fragmented BUSCOs, M = missing BUSCOs.

**Dataset**	**Species**	**BUSCO result**
Transcriptome	*Euroleon nostras*	C:86.0%[S:83.6%,D:2.4%],F:7.0%,M:7.0%
	Chrysoperla carnea	C:89.3%[S:88.2%,D:1.1%],F:4.8%,M:5.9%
Genome	*Euroleon nostras*	C:98.6%[S:96.1%,D:2.5%],F:0.5%,M:0.9%
