# Supplementary material for: Divergent venom effectors correlate with ecological niche in neuropteran predators
Source: Commun Biol. 2024 Aug 13;7:981. doi: 10.1038/s42003-024-06666-9 (PMC11319779; doi:10.1038/s42003-024-06666-9)
Supplement: Supplementary file 3 — Description of Additional Supplementary Files [file 42003_2024_6666_MOESM3_ESM.pdf]

# Description of Additional Supplementary Files

File: Supplementary Data 1

Description: Sequence information, functional annotations and expression values for venom protein candidates of Euroleon nostras identified by transcriptomic analysis. Source data for Figure 3.

File: Supplementary Data 2

Description: Sequence information, functional annotations and expression values for venom protein candidates of Chrysoperla carnea identified by transcriptomic analysis. Source data for Figure 3.

File: Supplementary Data 3

Description: Source data for Figure 6 (insecticidal assay).
