# Supplementary material for: Divergent venom effectors correlate with ecological niche in neuropteran predators
Source: Commun Biol. 2024 Aug 13;7:981. doi: 10.1038/s42003-024-06666-9 (PMC11319779; doi:10.1038/s42003-024-06666-9)
Supplement: Supplementary file 7 — Reporting Summary [file 42003_2024_6666_MOESM7_ESM.pdf]

nature portfolio

Corresponding author(s): Dr. Heiko Vogel

Last updated by author(s): Jun 6, 2024

Reporting Summary

Nature Portfolio wishes to improve the reproducibility of the work that we publish. This form provides structure for consistency and transparency in reporting. For further information on Nature Portfolio policies, see our Editorial Policies and the Editorial Policy Checklist.

Statistics

For all statistical analyses, confirm that the following items are present in the figure legend, table legend, main text, or Methods section.

n/a	Confirmed	
☐	☒	The exact sample size (*n*) for each experimental group/condition, given as a discrete number and unit of measurement
☐	☒	A statement on whether measurements were taken from distinct samples or whether the same sample was measured repeatedly
☐	☒	The statistical test(s) used AND whether they are one- or two-sided<br>*Only common tests should be described solely by name; describe more complex techniques in the Methods section.*
☐	☒	A description of all covariates tested
☒	☐	A description of any assumptions or corrections, such as tests of normality and adjustment for multiple comparisons
☒	☐	A full description of the statistical parameters including central tendency (e.g. means) or other basic estimates (e.g. regression coefficient) AND variation (e.g. standard deviation) or associated estimates of uncertainty (e.g. confidence intervals)
☒	☐	For null hypothesis testing, the test statistic (e.g. *F*, *t*, *r*) with confidence intervals, effect sizes, degrees of freedom and *P* value noted<br>*Give P values as exact values whenever suitable.*
☒	☐	For Bayesian analysis, information on the choice of priors and Markov chain Monte Carlo settings
☒	☐	For hierarchical and complex designs, identification of the appropriate level for tests and full reporting of outcomes
☒	☐	Estimates of effect sizes (e.g. Cohen's *d*, Pearson's *r*), indicating how they were calculated

Our web collection on statistics for biologists contains articles on many of the points above.

Software and code

Policy information about availability of computer code

Data collection	MassLynx v4.1, Leica Application Suite X
Data analysis	R v4.3.2, RStudio v2023.6.2.561 , MASCOT v2.6.0, MaxQuant v1.6.17.0, CLC Genomics Workbench v11.1, OmicsBox, BUSCO v3, ArrayStar v17, Hifiasm v0.16.0, BLAST v2.15.0, NRecon 2.0.0.5, Dragonfly 2022.2

For manuscripts utilizing custom algorithms or software that are central to the research but not yet described in published literature, software must be made available to editors and reviewers. We strongly encourage code deposition in a community repository (e.g. GitHub). See the Nature Portfolio guidelines for submitting code & software for further information.

Data

Policy information about availability of data

All manuscripts must include a data availability statement. This statement should provide the following information, where applicable:

- Accession codes, unique identifiers, or web links for publicly available datasets
- A description of any restrictions on data availability
- For clinical datasets or third party data, please ensure that the statement adheres to our policy

The proteomic data have been deposited in the ProteomeXchange Consortium (http://proteomecentral.proteomexchange.org) via the PRIDE partner repository with the dataset identifier PXD047026. The transcriptomic data have been deposited in the European Nucleotide Archive (ENA) at EMBL-EBI under accession

March 2021

number PRJEB70475 (https://www.ebi.ac.uk/ena/browser/view/PRJEB70475). The genomic dataset for E. nostras has been deposited in the Edmond data repository and can be accessed under the following weblink: https://doi.org/10.17617/3.YZVPXB.

Human research participants

Policy information about studies involving human research participants and Sex and Gender in Research.

Reporting on sex and gender	The study did not involve human research participants.
Population characteristics	The study did not involve human research participants.
Recruitment	The study did not involve human research participants.
Ethics oversight	The study did not involve human research participants.

Note that full information on the approval of the study protocol must also be provided in the manuscript.

Field-specific reporting

Please select the one below that is the best fit for your research. If you are not sure, read the appropriate sections before making your selection.

☒ Life sciences ☐ Behavioural & social sciences ☐ Ecological, evolutionary & environmental sciences

For a reference copy of the document with all sections, see nature.com/documents/nr-reporting-summary-flat.pdf

Life sciences study design

All studies must disclose on these points even when the disclosure is negative.

Sample size	For transcriptomic and proteomic analysis, pooled samples were analyzed. For the insecticidal assays, the sample size is given in the methods section.
Data exclusions	No data were excluded.
Replication	FISH and HCR-FISH analyses were performed with 3 and 2 replicates, respectively, to ensure reproducibility.
Randomization	The samples for all analyses were selected randomly.
Blinding	Blinding was not relevant for this study.

Reporting for specific materials, systems and methods

We require information from authors about some types of materials, experimental systems and methods used in many studies. Here, indicate whether each material, system or method listed is relevant to your study. If you are not sure if a list item applies to your research, read the appropriate section before selecting a response.

Materials & experimental systems

n/a	Involved in the study	
☒	☐	Antibodies
☒	☐	Eukaryotic cell lines
☒	☐	Palaeontology and archaeology
☐	☒	Animals and other organisms
☒	☐	Clinical data
☒	☐	Dual use research of concern

Methods

n/a	Involved in the study	
☒	☐	ChIP-seq
☒	☐	Flow cytometry
☒	☐	MRI-based neuroimaging

Animals and other research organisms

Policy information about studies involving animals; ARRIVE guidelines recommended for reporting animal research, and Sex and Gender in Research

Laboratory animals	Chrysoperla carnea, obtained from Katz Biotech AG (commercial laboratory rearing)

Wild animals	Euroleon nostras larvae were collected in the field in Jena, Germany.
Reporting on sex	Sex was not considered in the study design.
Field-collected samples	The larvae of Euroleon nostras were either used directly for the experiments or kept at room temperature in containers filled with clean glass beads. They were fed lepidopteran larvae once a week.
Ethics oversight	No ethical approval is required for experiments on insects.

Note that full information on the approval of the study protocol must also be provided in the manuscript.
